# National Trends in Hospitalizations for Appendectomy in Children, 2001-2017

**DOI:** 10.4274/TJAR.2025.241755

**Published:** 2025-05-30

**Authors:** Ludmilla Candido Santos, Jingya Gao, Ronaldo C. Fabiano Filho, Piero F. Mejia, Lacey B. Robinson, Carlos A. Camargo Jr.

**Affiliations:** 1 Massachusetts General Hospital, Department of Emergency Medicine, Boston, United States; 2 Massachusetts General Hospital Department of Medicine, Division of Rheumatology, Allergy and Immunology, Boston, United States; 3 Harvard Medical School, Boston, United States

**Keywords:** Appendicitis, cross-sectional studies, epidemiology, perioperative care, public health

## Abstract

**Objective:**

Appendectomy for acute appendicitis is the most common acute surgical procedure in children. Recent changes in appendicitis management have likely modified the nature and cost of hospitalizations for this condition.

**Methods:**

Using data from the National Inpatient Sample from 2001 to 2017, we performed a cross-sectional study and identified the temporal changes in hospitalization for appendectomy. Changes in relative hospitalization cost and length-of-stay were also studied to assess their associations with the changes in procedure incidence. Patient and hospital characteristics were considered to understand outcome disparities between groups. Geographic variation in the outcomes was also identified at the United States region and division levels.

**Results:**

The incidence of appendectomy hospitalization decreased from 11.2 to 6.4 per 10,000 person-years between 2001 and 2017. Conversely, the median procedure cost increased 61% during this same period. The temporal changes in appendectomy hospitalization varied according to patient and hospital characteristics, as well as geographic locations.

**Conclusion:**

The overall incidence of appendectomies in children decreased substantially from 2001 to 2017, yet the trend for costs was in the opposite direction. The data on the clinical factors driving these trends can be useful in guiding policies with evidence-based guidelines that help optimize clinical decisions and the effective use of resources in the management of appendicitis.

**Main Points**

• In this nationwide database analysis, we demonstrated the incidence of appendectomies among children decreased dramatically between 2001 and 2017.

• We found evidence, however, that associated costs are rising, suggesting that despite the reduction in incidence, modifications in the approach to appendicitis have made the procedure itself more expensive, even after appropriate cost adjustments.

• By improving the understanding of patterns of cost distribution, the described trends may help identify potential disparities in resource utilization in the management of acute appendicitis in children and provide data that could affect future research, practice, and health policy.

## Introduction

Pediatric hospitalizations accounted for approximately 15% (5.2 million) of all hospital stays in the United States (U.S.) in 2019, with an aggregate cost of more than $46 billion.^1^ Between 2010 and 2016, there was a 20% decrease in pediatric hospitalizations, but a rise in 30-day readmissions, with a higher proportion of chronic complex comorbidities.^2^ The annual pediatric hospitalization cost has increased over the years, largely due to the increase in the mean hospital costs per day, a rise particularly marked for surgical stays.^1, 2, 3, 4^

Hospitalization for acute appendicitis, the most common acute pediatric surgical condition,^1, 4^ decreased by 27% from 2000 to 2012.^1, 5^ During this time, total index pediatric admissions similarly decreased by 21% from 2000 to 2016.^1, 2^ Yet, pediatric healthcare expenses are growing faster than in other age groups.^6, 7^ Appendicitis-related hospitalizations cost approximately $3 billion a year within the U.S.,^2, 4^ and appendicitis remains the 5^th^ most common reason for hospitalizations among children.^3, 4^

Even though the improvements in pediatric health care reduce childhood morbidity and mortality,^8^ they do not come without a cost, and limited data are available about the continued trends in the surgical management of appendicitis, especially with the emerging non-surgical approach.^9^ Therefore, we investigated temporal trends in national inpatient appendectomies among children to better understand resource utilization and patterns of cost distribution, including potential disparities in the management of acute appendicitis in children. The use of data from 2001 to 2017 for our study brings a dataset that is representative of a stable pre-pandemic period, offering a more consistent and reliable baseline for analysis compared to more recent data. The coronavirus disease-2019 pandemic significantly disrupted healthcare systems and patient behaviors, potentially skewing data on hospital admissions and surgical procedures. Additionally, the extensive timespan of our dataset allows for the identification of long-term trends and patterns in appendectomy practices. 

## Methods

This study was developed with de-identified, publicly available national data. Local ethics committee review was waived.

### Study Design, Data Source, and Study Population

We performed a cross-sectional study of appendectomy hospitalizations in pediatric patients using the 2001-2017 National Inpatient Sample (NIS) data. NIS is the largest publicly available database for all-payer national inpatient care, developed by the Healthcare Cost and Utilization Project (HCUP). The HCUP divides the U.S. into nine geographic regions.^10^ The database is designed to analyze inpatient utilization, access, cost, quality, and outcomes. Before 2012, the database retained all discharge data from a 20% sample of the hospitals in the U.S., stratified by region, hospital location, teaching status, ownership, and hospital size. Beginning in 2012, NIS sampled approximately 20% of discharge data from all HCUP-participating hospitals, excluding rehabilitation and long-term acute care hospitals. A NIS trend weight was applied to our analysis; thus, the estimates are comparable across the study years.^10^

**Figure 1:**
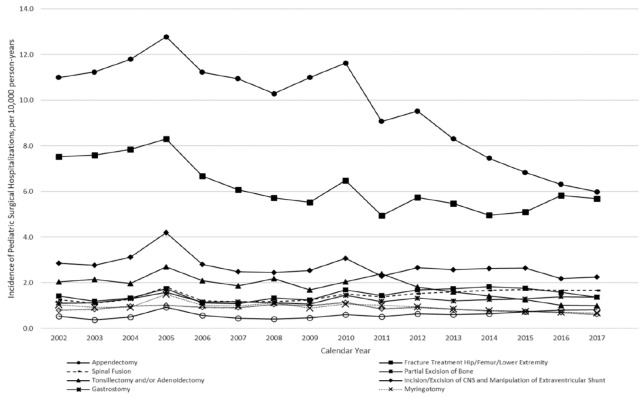
Incidence of pediatric surgical hospitalizations per 10,000 person-years, 2001-2017.

Our study focused on pediatric patients (age <18 years) hospitalized for appendectomy between 2001 and 2017. The hospitalization data for the population was then compared with hospitalizations for other common pediatric surgical procedures (Figure 1). A population with appendicitis diagnosis, (with and without appendectomy) was specified separately to understand its impact on appendectomy hospitalization within the study period. Procedures and diagnoses were identified by Clinical Classifications Software (CCS), which collapses the International Classification of Disease (ICD) codes into clinically meaningful categories (see detailed CCS groups in Supplementary Table 1). NIS switched from the 9^th^ version of the ICD codes (ICD-9) to the 10^th^ version (ICD-10) to identify diagnoses and procedures in October 2015. A new scheme was implemented in CCS to accommodate this change. The 1^st^ three CCS groups were reviewed to capture appendicitis diagnosis and the procedures of interest.

### Outcome

The primary outcome was the incidence of appendectomy among hospitalized pediatric patients. Any appendicitis hospitalizations without appendectomy procedures were defined as non-surgical cases. The secondary outcomes included hospitalization costs and hospital length-of-stay for appendectomy. Hospital costs were converted from hospital charges using the cost-to-charge ratio provided by NIS.^11^ All hospital costs were adjusted to 2017 U.S. dollars using the consumer price index to facilitate the comparison of costs across years.^12^ Costs reflect the actual expenses incurred in the process of delivering hospital services, including wages, supplies, and utility costs.

### Other Factors

Patient and hospital characteristics were evaluated to assess their associations with the temporal trend of appendectomy hospitalization. Patient demographic factors included age, sex, race, and ethnicity. Hospital factors included geographic locations (in regions and in divisions), urbanicity, teaching status, and hospital bed size (small, medium, and large). As NIS sampling was stratified by census region instead of census division before 2012, geographic estimates were reported in regions (Northeast, Midwest, South, and West) in Table 1 to cover the entire study period. Hospital bed size categories are specific to the hospital’s region and teaching status. Rural and urban non-teaching hospitals were combined and compared with urban teaching hospitals to identify potential disparities in hospitalization cost and length-of-stay related to the hospitals’ teaching status.^13^

### Statistical Analysis

All analyses were performed with statistical analysis system (SAS) software, version 9.4 (SAS; Cary, NC). The estimates were calculated using appropriate survey commands in SAS and sample weights provided by NIS. Weighted percentages were reported with a 95% confidence interval. Continuous outcomes were reported as medians with interquartile range. To stabilize estimates, the descriptive results were grouped as 2001-2002, 2003-2005, 2006-2008, 2009-2011, 2012-2014, and 2015-2017. We performed Rao-Scott chi-square test to assess the temporal trend of appendectomy hospitalization, and a linear regression to examine the changes in procedure cost and hospital length of stay. A two-tailed *P* value < 0.05 was considered statistically significant.

The annual incidence of pediatric hospitalizations was calculated by dividing the estimated number of hospitalizations by the pediatric population from the U.S. Census Bureau.^14^ The annual incidence trends were then calculated for appendectomy and other common pediatric surgical procedures. We also examined the incidence trend of hospitalizations diagnosed with appendicitis, grouped by treatment type. The median cost of the appendectomy procedure was calculated in the overall population and stratified by teaching status. An interaction term of linear regression was tested between the year variable and the teaching variable to determine the difference in temporal change in hospitalization cost according to teaching status.

To understand the geographic variation in the outcomes, we mapped appendectomy incidences and hospitalization costs at the division level, as defined by U.S. Census Bureau.^14^ Due to NIS sampling strategy changes in 2012, all maps presented division-level outcomes in the earliest available year (2012) and the percentage change in 2017.

## Results

### Temporal Trends in Overall Incidence of Appendectomy

**Figure 2:**
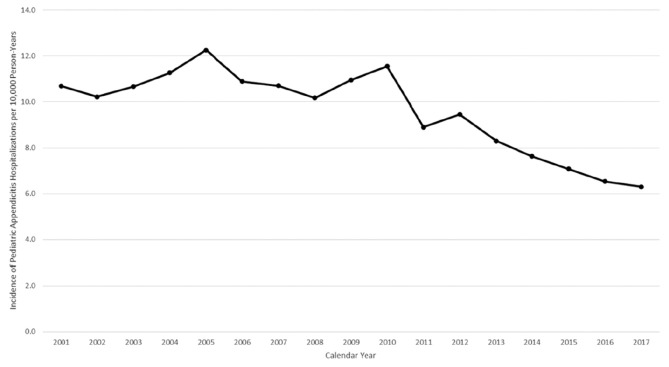
Incidence of pediatric appendicitis diagnosed hospitalizations per 10,000 person-years, 2001-2017

**Figure 3:**
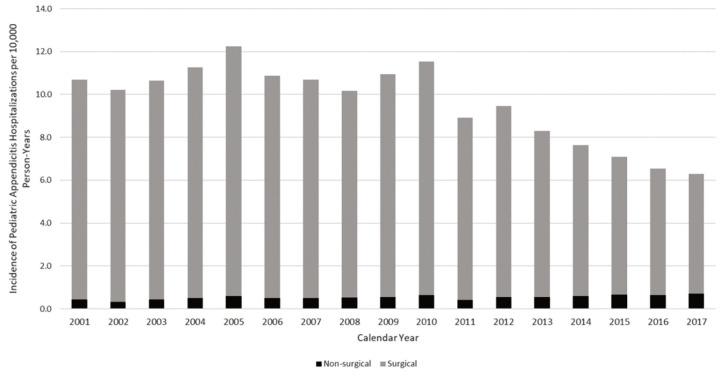
Incidence of pediatric appendicitis diagnosed hospitalizations by procedure type, 2001-2017

Overall, we identified 101,581 sampled hospitalizations for appendectomy, representing 256,783 visits. The incidence of appendectomy, the most frequent pediatric surgical procedure, decreased the most compared to other common surgeries, dropping from 11.2 to 6.4 per 10,000 person-years between 2001 and 2017 (Figure 1). A similar pattern was observed for the incidence of appendicitis hospitalization, which decreased from 10.7 to 6.3 per 10,000 person-years (Figure 2). The weighted estimates of non-operative hospitalizations for appendicitis diagnosis appeared stable across the study years (Figure 3).

### Trends in Appendectomy Incidence Across the U.S.

**Figure 4:**
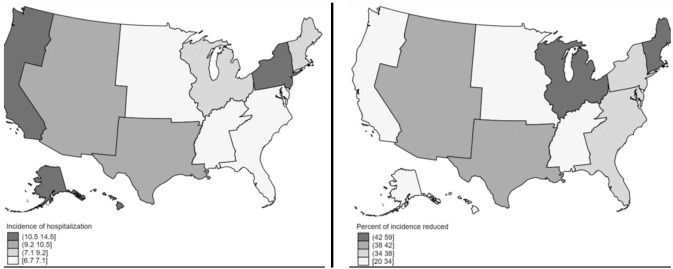
Incidence of pediatric appendectomy hospitalization per division in 2012 (left) and percent of incidence reduced between 2012 and 2017 (right)

While appendectomy incidence declined across the U.S., New England experienced the largest decrease of -59% between 2012 and 2017 (Figure 4) (Supplementary Table 2). The incidence in East South Central (-20%), Pacific (-31%), and West North Central (-34%) experienced smaller but still substantial declines. The incidence of appendectomy in the Pacific division was the highest among all divisions in both 2012 and 2017.

### Temporal Trends in Hospital Costs for Appendectomy

**Figure 5:**
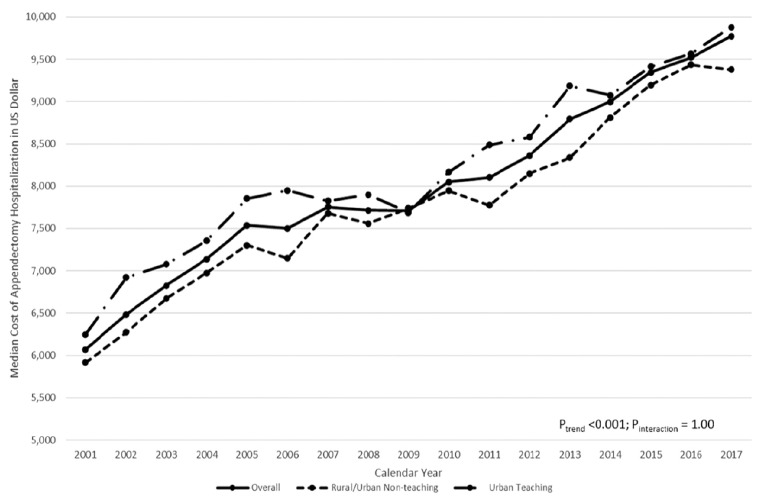
Median cost of pediatric appendectomy hospitalization in United States dollar, by hospital teaching status, 2001-2017

The median hospital cost for appendectomy significantly increased between 2001 and 2017 (*P_trend_*< 0.001, Figure 5), regardless of hospital location or teaching status. The non-significant interaction term (*P_interaction_*=1.00) indicated a similar increase in appendectomy cost between urban teaching and rural/urban non-teaching hospitals. Additionally, the overall median length-of-stay for appendectomy hospitalizations was unchanged from 2001 to 2017.

**Figure 6:**
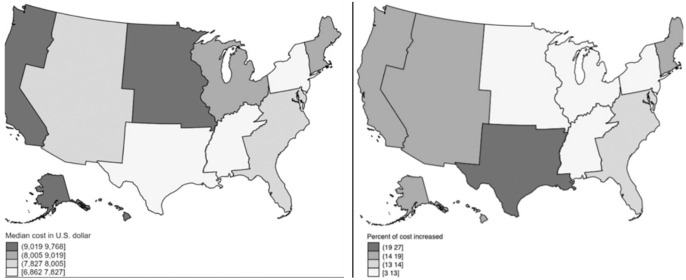
Median cost of appendectomy hospitalization per division in 2012 (left) and percent of cost increased between 2012 and 2017 (right)

From 2012 to 2017, the median hospital cost for appendectomy in West South Central increased by 27% (Figure 6), which was higher than in other divisions. The median cost in East South Central stayed relatively stable. With the highest appendectomy incidence, the median hospitalization cost in the Pacific division was also greater than other divisions in 2012 and 2017 ($9,768 and $11,589, respectively; Supplementary Table 2).

### Patient and Hospital Characteristics for Appendectomy Hospitalizations

The median age of pediatric appendectomy hospitalizations decreased slightly from 12 to 11 years over the study period (*P* < 0.001, Table 1). The number of hospitalizations varied by race/ethnicity. The percentage of Hispanic patients significantly increased from 20% to 36% between 2001 and 2017 (*P_trend_* < 0.001).

We also observed variations in temporal hospitalization trends by hospital characteristics. The number of patients admitted for appendectomy to rural or urban non-teaching hospitals decreased during the study period (*P_trend_* < 0.001). Meanwhile, the percentage of appendectomy hospitalizations in urban teaching hospitals increased from 44% to 76% (*P_trend_* < 0.001). The proportion of appendectomy hospitalizations from the West region was significantly increased (*P_trend_*=0.004), while the proportion of hospitalizations from the Midwest region was significantly decreased during the same period (*P_trend_*=0.01). Additionally, hospitalizations for appendectomy occurred most often in hospitals with a large number of beds and significantly increased across years (*P_trend_*=0.03).

## Discussion

The results of this multi-year, cross-sectional study indicate that the incidence of appendectomy decreased markedly across the U.S., from 11.2 to 6.4 per 10,000 person-years between 2001 and 2017. There was a similar decline in the incidence of hospitalization for acute appendicitis. The observed reduction in appendectomy incidence may be partially attributed to the increased adoption of advanced diagnostic protocols and imaging techniques, which have improved diagnostic accuracy and potentially reduced unnecessary surgical interventions.^15, 16, 17, 18^ We identified differences across sociodemographic groups and divisions in the country that may reflect variability in access to or utilization of healthcare.^1, 5, 7^ Despite a decline in overall appendectomies over this period, we observed an increase in costs. While our study did not specifically analyze individual contributors to cost increases, we acknowledge that the trends in increased costs are likely multifactorial. Several potential causes are suggested, including the shift towards laparoscopic procedures, which may have higher initial costs but offer benefits in terms of recovery and complications. The increased use of advanced diagnostic imaging, such as computed tomography (CT) scans, has improved diagnostic accuracy but also contributed to higher overall costs. The more widespread use of antibiotics, both pre- and post-operatively, has also been noted as a factor in cost escalation. Furthermore, studies have identified disparities in healthcare utilization and costs between urban and rural settings, which may influence overall expenditure trends. It’s also worth noting that trainee involvement in surgeries, while crucial for medical education, can increase operative time and subsequently affect costs.^15^

Appendectomy remains by far the most common surgical procedure performed among U.S. children in the inpatient setting. We highlight the considerable decrease in its incidence from 2001 to 2017, while the incidence remained roughly stable for other procedures within the same timeframe (Figure 1). This trend can be at least partially attributed to modifications in the diagnosis and management of acute appendicitis in children.^16, 17^

Specifically, acute appendicitis management has evolved to include more conservative approaches, such as antibiotic therapy, before operative resection, unless in severe or unstable cases,^18^ contributing to the decrease in the appendectomy rate.^16^ Classically, acute appendicitis in children was an indication for appendectomy,^17^ which reduces the rate of associated complications, especially appendix perforation.^16, 17^ However, not all appendicitis cases have similar risks of complication if not promptly treated surgically.^19^ Indeed, studies have demonstrated that some pediatric patients, particularly those over the age of 5 years with uncomplicated acute appendicitis within 48 hours of symptom onset, could be reasonably treated with conservative antibiotic therapy with a successful treatment rate above 50%.^16, 17^ Moreover, because uncomplicated acute appendicitis cases are much more common than complicated ones, our results may suggest that a non-operative approach could have been considered to a relatively high number of affected patients, resulting in a downwards trend in the incidence of appendectomy.^16^ Meanwhile, some authors have hypothesized reasons for the decreasing trend seen in appendectomies, such as technological changes in diagnostic and treatment techniques, management of complications, implementation of new strategies and protocols, or associations with chronic conditions.^15^

Historically, acute appendicitis has been a clinical diagnosis based on typical abdominal pain features and associated signs and symptoms during physical examination and patient history.^20^ Further laboratory evaluation commonly demonstrates leukocytosis, elevated absolute neutrophil count, and C-reactive protein, which have variable performance and predictive values across studies.^21^ To increase diagnostic accuracy, tools such as the Pediatric Appendicitis Score (PAS) and the Alvarado score are used as a combination of these variables and provide valuable clinical utility.^21, 22^ Concurrently, readily available, sensitive imaging methods, ultrasound, and CT scans have emerged as diagnostic tools. These tools increase the reliability of diagnosis and help identify cases and complications of acute appendicitis.^23^

The recent efforts toward non-surgical approaches to appendicitis may also play a role in an increase in hospital length-of-stay (Table 1). Despite the constant rate of non-surgical management of appendicitis over the years, this approach represented a higher proportion compared to a surgical approach, as the overall frequency of these hospitalizations declined. Conservative management could result in longer hospitalizations.^24^ When a non-operative approach is proposed, antibiotics are initially administered intravenously in the inpatient setting, and are eventually transitioned to oral medication, with the patient being discharged when feasible.^16, 25^ Since a conservative approach is not currently recommended by guidelines,^26^ clinical reasoning for uncomplicated cases tends to require longer observation periods, with additional imaging and laboratory confirmatory tests increasing the length of stay in the hospital days in the hospital for intravenous antibiotics.

Studies involving adults have demonstrated a similar pattern of overall longer hospital stays for cases treated conservatively, due to variable rates of failure of antibiotic therapy, complications, and reoperation.^16, 24, 25^ Furthermore, approximately one-third of patients initially assigned to receive antibiotic therapy undergo an appendectomy afterwards.^18^ Regardless of age, laparoscopic techniques may safely manage uncomplicated appendicitis; however, when complications develop, conventional open techniques tend to be the first choice, and these are associated with longer recovery periods, delayed return to normal activities, and more pronounced psychological effects of hospitalization.^16, 17, 24, 25, 26^

Our nationwide data are also consistent with the previously described decrease in the incidence of appendicitis since the 1940s, with a 14.6% national decrease in the rate observed between 1970 and 1984.^27^ Even though a similar nationwide decrease in appendectomies continues to occur, racial and geographic variability seems to play a role in the uneven distribution of this procedure across different regions of the U.S.^28^ Disparities in health outcomes have been shown to be affected by many variables beyond the healthcare setting,^28, 29^ race, income, healthcare access and utilization may influence not only when patients will seek care for one specific condition, but also the success of treatment, rate of complications, availability of follow-up, and overall mortality.^28, 30^

For acute appendicitis, many experts have highlighted how crucial it is to ensure that no patients, especially vulnerable populations, are deprived of necessary care, as this can result in an increased likelihood of unfavorable outcomes.^18^ This population commonly relies on community hospitals, frequently located in rural areas,^28, 30^ and such healthcare settings typically have less resources available when compared to urban-teaching hospitals.^13^ The increase in costs of appendectomy over the years, in both healthcare settings, illustrates that high-quality, cost-effective management could help optimize resource utilization and lessen the important healthcare burden represented by appendicitis.^2, 4^ An overall rise in health care costs over the study period most likely reflects the constant incorporation of new medicines, procedures, and technologies.^21, 23^ With the increasing concern about the affordability of health care, the trends provide an important overview of the incidence and spending associated with the most common emergent surgical procedure in the pediatric population.

### Study Limitations

This study comes from the analysis of a large nationally representative database over a long study period. Despite the widely generalizable results, these trends presented here were obtained based on procedure codes, which is a system prone to error, and which can lead to misclassification of the outcome. Considering the study period, we also need to acknowledge that administrative data transitioned from ICD-9 to ICD-10 codes in October 2015, which may affect trends, and that there were data-structure changes in NIS elements that occurred in the same year.^10, 11^ Furthermore, NIS implemented a sample redesign in 2012 to better represent U.S. hospitals. As the old design was stratified by regions instead of divisions, it would be inaccurate to calculate division-level estimates before 2012. Therefore, the maps in our study focused on the change in incidence in the later years by division. Additionally, some secondary diagnoses codes might still refer to surgical inpatient outcomes but do not necessarily differentiate between complications and comorbid conditions, limiting our ability to specify costs related to the main procedure.

## Conclusion

Between 2001 and 2017, we identified a significant decline in the incidence of appendectomies among U.S. children and overall hospitalizations for acute appendicitis. By contrast, costs for hospitalizations for appendectomy have increased, suggesting that modifications in the approach to appendicitis, including utilities and supplies used in its management, have made the procedure itself more expensive, even after adjusting for inflation. The differences in patient characteristics and geographic distribution of the incidence of this procedure emphasize the multitude of components involved in the surgical approach to appendicitis. Future analyses of clinical data could consider whether the increase in costs translates into better outcomes such as fewer complications and readmissions, aiming to provide improvements in care for patients with appendicitis. Previous data have shown that access to healthcare, and ultimately outcomes, vary considerably by sociodemographic factors, and these aspects are useful in generating evidence-based guidelines valuable in the evaluation and management of appendicitis. Our findings emphasize the importance of standardized care protocols to reduce variability and improve outcomes, while also suggesting that lessons from international practices, such as broader adoption of non-operative management, could further enhance pediatric appendicitis care in the U.S. Finally, the overall rising costs and disparities seen in the approach to appendicitis should call for the development of treatment strategies and policies that consider clinical factors in the decision-making process of managing pediatric appendicitis.

## Ethics

**Ethics Committee Approval:** Waived (analysis of de-identified, publicly available national dataset).

**Informed Consent:** Not applicable.

## Supplementary Materials

Supplementary Table 1 and 2 https://l24.im/Hn60ohB

## Figures and Tables

**Table 1. Patient and Hospital Characteristics of Appendectomy Hospitalizations in the United States Children, 2001-2017:** 

**-**	**2001-2002**	**2003-2005**	**2006-2008**	**2009-2011**	**2012-2014**	**2015-2017**	**Overall weighted frequency**	***P_trend_***
**-**	**Weighted percentage (95% CI)**	**Weighted percentage (95% CI)**	**Weighted percentage (95% CI)**	**Weighted percentage (95% CI)**	**Weighted percentage (95% CI)**	**Weighted percentage (95% CI)**		
**Age, median (IQR)**	12 (8-15)	12 (8-15)	12 (8-15)	12 (8-15)	11 (8-14)	11 (7-14)	13,463,915	< 0.001
**Female, sex**	40.7 (40.1-41.3)	40.7 (40.3-41.1)	40.1 (39.7-40.6)	40.7 (40.2-41.2)	40.7 (40.2-41.3)	40.2 (39.6-40.8)	487,155	0.28
**Race/ethnicity**								
Non-hispanic white	45 (42-48)	41 (38-44)	42 (39-45)	45 (42-48)	45 (44-47)	42 (41-44)	531,773	0.053
Non-hispanic black	5 (4-6)	5 (4-6)	5 (4-6)	5 (4-6)	5.9 (5.5-6.3)	6.2 (5.7-6.6)	64,603	0.01
Hispanic	20 (17-23)	19 (16-22)	19 (16-21)	27 (24-30)	34 (32-36)	36 (34-38)	306,535	< 0.001
Asian, Pacific Islander, or Native American	2.3 (1.8-2.8)	1.8 (1.4-2.2)	2.2 (1.8-2.5)	2.9 (2.3-3.4)	3.4 (3.1-3.7)	3.6 (3.3-4)	31,996	< 0.001
Other	3.5 (2.6-4.4)	3 (2-4)	3.4 (2.8-4)	4 (3-5)	4.9 (4.5-5.3)	5.2 (4.7-5.7)	47,805	< 0.001
Missing	25 (21-28)	30 (26-34)	28 (25-32)	16 (13-19)	6 (5-8)	6 (5-8)	247,783	< 0.001
**Hispanic**								
Yes	20 (17-23)	19 (16-22)	19 (16-21)	27 (24-30)	34 (32-36)	36 (34-38)	306,535	< 0.001
No	55 (52-59)	51 (47-54)	53 (50-56)	57 (53-60)	59 (58-61)	57 (55-59)	676,177	< 0.001
Missing	25 (21-28)	30 (26-34)	28 (25-32)	16 (13-19)	6 (5-8)	6 (5-8)	247,783	< 0.001
**Length of stay per hospitalization, median (IQR)**	2 (1-4)	2 (1-4)	2 (1-4)	2 (1-4)	2 (1-4)	2 (1-5)	4,375,534	< 0.001
Urban teaching	2 (1-5)	2 (1-5)	2 (1-5)	2 (1-4)	2 (1-5)	3 (1-5)	1,227,269	0.50
Rural/urban non-teaching	2 (1-3)	2 (1-3)	2 (1-3)	2 (1-3)	2 (1-3)	2 (1-4)	104,375	0.14
**Location/teaching status**								
Rural	18 (15-20)	14 (12-16)	13 (11-15)	11 (9-13)	8.7 (8.1-9.4)	6.2 (5.6-6.7)	147,559	< 0.001
Urban non-teaching	39 (35-43)	40 (35-44)	39 (35-43)	35 (30-40)	27 (25-29)	17 (16-19)	418,301	< 0.001
Urban teaching	44 (38-49)	46 (41-51)	48 (43-53)	54 (48-60)	64 (62-66)	76 (75-78)	661,409	< 0.001
**Region**								
Northeast	19 (15-22)	19 (16-22)	20 (16-24)	16 (12-20)	17 (15-19)	16 (14-18)	221,620	0.30
Midwest	22 (19-26)	20 (16-24)	20 (17-22)	17 (13-20)	16 (15-18)	15 (14-17)	226,701	0.01
South	33 (28-37)	34 (30-39)	33 (28-38)	34 (28-40)	33 (30-36)	34 (32-37)	414,184	0.98
West	26 (22-31)	26 (22-31)	27 (23-31)	33 (27-39)	34 (31-37)	34 (31-37)	367,991	0.004
**Bed size**								
Small	15 (12-19)	14 (11-17)	15 (12-18)	11 (8-13)	14 (13-16)	16 (14-18)	173,641	0.03
Medium	31 (26-36)	30 (25-34)	25 (21-28)	25 (20-31)	25 (23-28)	25 (22-28)	329,824	0.09
Large	53 (49-58)	56 (51-61)	60 (56-65)	64 (58-69)	60 (58-63)	59 (56-62)	723,804	0.03

CI, confidence interval; IQR, interquartile range
